# Microstructure and Tribological Properties of Laser Forming Repaired 34CrNiMo6 Steel

**DOI:** 10.3390/ma11091722

**Published:** 2018-09-14

**Authors:** Chunping Huang, Xin Lin, Haiou Yang, Fencheng Liu, Weidong Huang

**Affiliations:** 1 State Key Laboratory of Solidification Processing, Northwestern Polytechnical University, Xi’an 710072, China; hcp98106@163.com (C.H.); huang@nwpu.edu.cn (W.H.); 2 Engineering Research Center in Additive Manufacturing, Nanchang Hangkong University, Nanchang 330063, China; fencheng999@nchu.edu.cn

**Keywords:** laser forming repair, quenching and tempering steel, microstructure, tribological property

## Abstract

Laser forming repair (LFR) technology has considerable potential in high strength steel structure repair. 34CrNiMo6 steel has been widely used in high-value components, and it is imperative to repair these damaged components. In this study, two different thicknesses of repaired layers are deposited on the 34CrNiMo6 wrought substrate with five layers and 20 layers via LFR technology. The microstructure, phases, microhardness, and tribological properties are analyzed using optical microscopy, scanning electron microscopy, X-ray diffraction, Vickers hardness testing, and dry sliding wear testing. These results show that the 34CrNiMo6 repaired layers were successfully deposited on the substrate. The microstructure of the laser-repaired layers in the five-layer sample included bainite and retained austenite. For the 20-layer sample, the microstructure in the top of the repaired layers was still bainite and retained austenite, whereas that in the bottom of the repaired layers was transformed into ferrite and cementite. The average coefficients of friction of repaired layers is not significantly different from the substrate. The wear rate of the five LFR layers, 20-layer LFR, and substrate samples were 12.89 × 10^−6^, 15 × 10^−6^, and 23.87 × 10^−6^ mm^3^/N·m, respectively. The laser forming repaired samples had better wear resistance compared to the substrate. The wear mechanism of laser forming repaired samples is abrasive wear; whereas that of the substrate is abrasive wear and fatigue wear.

## 1. Introduction

The Cr-Ni-Mo steels, such as 34CrNiMo6, AISI 4340, and 5CrNiMo, have high strength, good ductility, and excellent corrosion resistance after quenching and tempering (QT) [1]. The 34CrNiMo6 (material No. 1.6582) is a typical quenched and tempered Cr-Ni-Mo steel that can be used where excellent fatigue and wear properties are required. Typical products include propeller shafts, gear shafts, and discs in aircraft [2,3]. Severe failures may happen when in use. The wear-related problems are common initiators of failure for high cost shafts used in the turbine industry. Therefore, these worn-out high cost components must be repair to extend the service life.

Conventional repair processes, such as thermal spraying [4], plasma spraying [5], and traditional arc welding [6], have been investigated for years to solve these problems. However, these repair techniques still have many shortcomings, such as a large heat affected zone and large deformation, all due to the high energy input from a large sized heat source. Laser forming repair (LFR) has the advantage of both confined and controlled heat input. Therefore, it is especially beneficial for the low-cost repair of damaged high-value components [7,8].

Laser forming repair, also known as laser additive manufacturing, owing to its cost-saving potential and the fine microstructures obtained due to the high cooling rate, is an advanced fabrication technology based on a new manufacturing principle of additive materials. It is gaining popularity as a rapid prototyping and repairing technology for manufacturing compact metallic components with high performance [9,10]. Attar et al. [11] investigated the difference between laser engineered net shaping (LENS) and selective laser melting (SLM), which showed that significantly higher laser power and energy density is required in LENS compared to SLM [12]. In the early years, laser melt particle injection (LMPI) was another technology that used laser-melted particles for surface treatment. Processes based on the fusion of applied powder particles to a substrate, using a continuous CO_2_ laser beam as the heat source, produce a variety of novel cladding [13,14]. The in situ function gradient coating possessing oxidation and corrosion resistance was fabricated on the surface by laser melt injection of nano-CeO_2_ [15]. Wang et al. [16] investigated the effect of electric-magnetic synergy on the reinforcement phase distribution in laser melt injection.

Many test results showed that satisfactory mechanical properties, such as yield strength (YS), ultimate strength (UTS), and hardness can be obtained by laser additive manufacturing for various types of steels [17,18,19,20,21,22,23,24]. Many valuable results have been obtained in Cr-Ni-Mo steel via laser additive manufacturing. Chen et al. [17] investigated the microstructure and tensile properties of 5CrNiMo steel by selective laser melting, which showed that the tensile strength and ductility of SLM samples were expected to be favorable given the lath-shaped martensite microstructure. Bhattacharya et al. [18] investigated the microstructure of AISI 4340 steel during direct metal deposition (DMD), which showed that the microstructure of the clad layer consist of ferrite, martensite, and cementite. Sun et al. [19] reported the influence of processing parameters on the lattice parameters of DMD AISI 4340 steel and indicated that martensite lattice parameters increased, while austenite lattice parameters decreased, with decreasing laser specific energy. Sun et al. [20] conducted systematic and more realistic engineering research on DMD AISI 4340 steel blocks, which included the evaluation of defect density, microstructure, hardness, and strength of as-deposited specimens. The microstructure included martensite and retained austenite. Tempering leads to the formation of tempered martensite. Previous studies [21,22] reported that the tempering temperature has a noticeable effect on the microstructure of 34CrNiMo6 steel during the heating process.

With this technique, different steels have been successfully deposited for repair of the substrate. Sun et al. [8] repaired HSLA-100 substrates with HSLA-100 powder by laser deposition and investigated the microstructure, hardness, and tensile property of repaired samples. Liu et al. [25] repaired 300M steel by laser forming repairs and investigated the microstructure, hardness, and tensile properties of repaired samples. Lin et al. [26] repaired 17-4PH stainless steel by laser forming repair and investigated the microstructure, hardness, and tensile properties of the repaired samples. Sun et al. [27] investigated the effect of laser clad repair on the fatigue behavior of AISI 4340 steel.

One of the most important causes of the shaft parts suffering damage and fail is wear. The study of tribological properties is also important for applications of laser forming repairs of shaft parts. However, to date, there have been no publications focusing on the effect of laser forming repair on the tribological properties of Cr-Ni-Mo steels. Generally, the wear resistance of a given material has a close relationship with its microstructure or forming process. So, studies on the effects of microstructure or forming process on the wear resistance are of important engineering value. In this study, the microstructure evolutions and hardness of LFR 34CrNiMo6 steel with two kinds of samples were analyzed. The phase and tribological properties of the last laser-repaired layer were investigated in detail. In particular, the micrographs of the sub-surface of the laser-repaired layers before and after a wear test were used to investigate the microstructural change in the wear process. This study provides essential results for the overall potential of laser forming repair as a future repair solution for shafts.

## 2. Experiment

**Figure 1:**
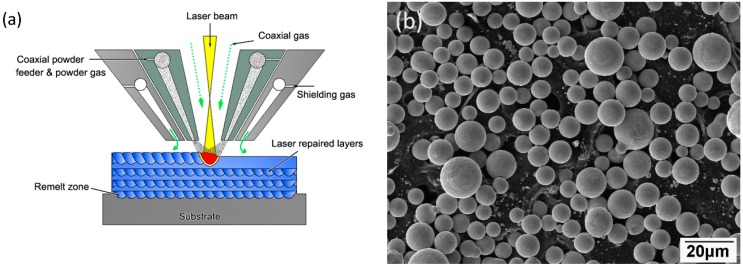
(**a**) Illustration of laser forming repair process and (**b**) scanning electron microscopy (SEM) micrographs of the as-received 34CrNiMo6 steel powder.

LFR samples of 34CrNiMo6 steels were formed on a LFR system, which included a 4 kW continuous wave CO_2_ laser, five-axis numerical control working table, powder feeder, and coaxial nozzle. Figure 1a depicts the LFR process. Continuously flowing argon gas was used to prevent the melt pool and the heat affected zone (HAZ) from oxidation and contamination during the LFR process. In the present experiment, 34CrNiMo6 alloy spherical powder with a nominal powder size of less than 150 μm was used (Figure 1b). Wrought 34CrNiMo6 steel plate was used as the substrate. The substrate surface was ground with SiC paper and cleaned with acetone prior to LFR. The chemical compositions (wt %) of 34CrNiMo6 powder are listed in Table 1. The powder was oven dried for 2 h at 150 °C in a vacuum drying furnace. The processing parameters of LFR are listed in Table 2. Notably, adjacent tracks in identical layers were scanned using reverse directional scan vectors and the layers were deposited in a crosshatched pattern.

LFR samples for microstructure observation were machined using a wire electrolytic-discharge machine. The microstructures of the samples were revealed using an etchant of 4% nitric acid alcohol solution for 10–15 s. The microstructure and phase constitution of LFR samples were examined using optical microscopy (OM, Olympus-GX71, Tokyo, Japan), scanning electron microscopy (SEM, Tescan mira3, Brno, Czech Republic), and X-ray diffraction (XRD, Philips X’Pert MPD PRO, Amsterdam, The Netherlands). SEM micrographs were taken both before and after wear tests to examine the microstructure. The XRD analysis was performed using a Philips X’Pert MPD PRO X-ray diffractometer with monochromatic CuKα radiation with 50 KV and 50 mA. XRD analyses were carried out on the sub-surface of the laser repaired samples with the intention of studying the phase of the worn surfaces. The microhardness was measured using a Vickers tester (Struers Duramin-A 300, Ballerup, Denmark) with a load of 200 g and loading time of 15 s. After the surface of the laser forming repaired samples was ground and polished to a distance of 0.2 mm from the surface, the wear test was performed. The worn surface microhardness tests were performed on all the wear test samples.

**Figure 2:**
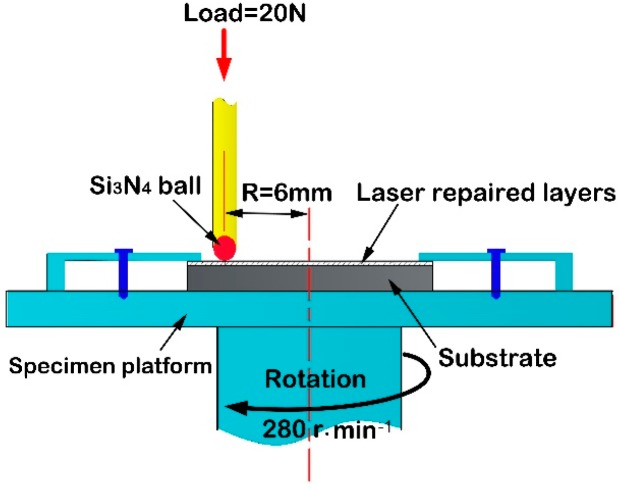
Schematic of wear test.

Wear tests on the laser forming repaired samples were conducted using a conventional ball-on disk tribometer (HT-1000, Zhongke Kaihua Technology Development Co., Ltd, Lanzhou, China) shown in Figure 2. The tribometer is a specialized pin-on-disk apparatus that can measure in situ friction and normal load data. It consists of a rotating/oscillating lower spindle on which the flat specimen is rigidly mounted, and a special top holder that applies a normal load in a controlled closed loop manner via an electromagnet. The ball specimen is mounted in a tubular collet assembly that is held stationary in a two-axis force transducer. The experiments were performed under laboratory conditions of 25 °C and 40% relative humidity (RH). For the ball-on-disk tests, the wear data were collected after 60 min. A constant normal force of 20 N was applied for all tests. The counter-body was a Si_3_N_4_ ball with a diameter of 4.75 mm and a hardness of 1500 HV. The tests were performed at 280 rpm. The weight losses of the wear specimens were determined by weighing the specimens before and after the wear process with an electronic balance.

## 3. Results and Discussion

### 3.1. Microstructure Evolution

**Figure 3:**
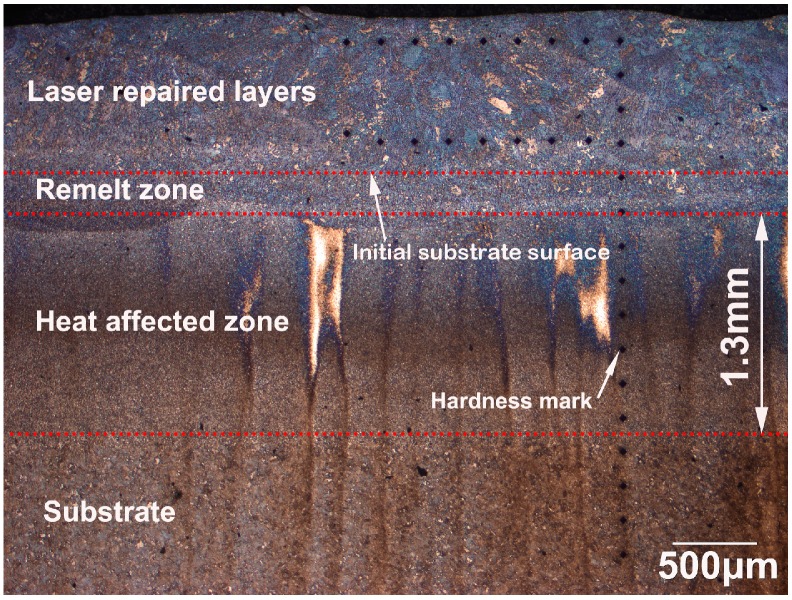
The cross-section of the top region in the five-layer laser forming repair (LFR) sample.

**Figure 4:**
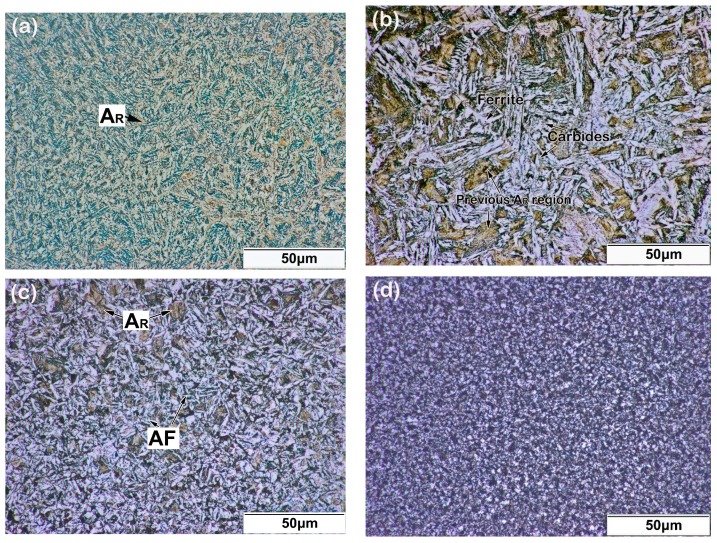
Microstructure of different areas in a five-layer LFR sample: (**a**) top of the laser repaired layers; (**b**) the remelt zone; (**c**) top of the heat-affected zone (HAZ); and (**d**) bottom of the HAZ.

Figure 3 shows the cross-section of the top region in a five LFR layer sample, which included laser repaired layers, remelt zone, heat affected zone (HAZ), and substrate. After the LFR process, the initial surface of the substrate was completely re-melted, and no defect, such as lack-of-fusion porosity, or crack, were found on the cross-section of the five LFR layer sample. We inferred that the thickness of the five LFR layers was approximately 0.86 mm, whereas the width of the heat affected zone was approximately 1.3 mm. The optical microscope (OM) micrographs shown in Figure 4 represent the microstructure evolution that occurred during the LFR process. As can be observed from Figure 4a, the microstructure of the last LFR layer was characterized by mostly bainite (including olive-colored bainite carbides and white bainite ferrite matrix) and network retained austenite (A_R_, brown phase). A detailed view of the microstructure in the last LFR layer can be observed by SEM in Section 3.3. The microstructure of the remelt zone (Figure 4b) mostly included ferrite, carbides, and a small amount of retained austenite. Most of the retained austenite was transformed into ferrite and cementite. The density of cementite in the previous A_R_ region was significantly higher than that of other regions. The microstructure in the top region of the HAZ is shown in Figure 4c, which included acicular ferrite (AF), carbides, and retained austenite. However, the microstructure in the bottom region of HAZ included fine ferrite and carbides. The grain size of ferrite, as shown in Figure 4d, was approximately 3 μm.

**Figure 5:**
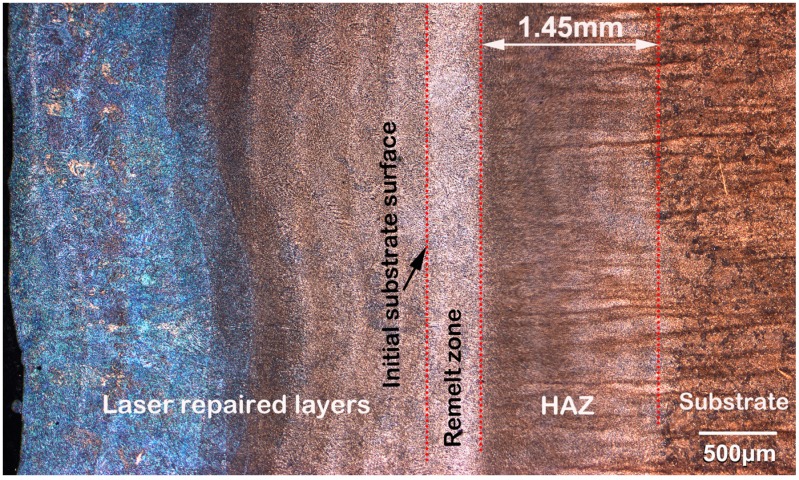
The cross-section of the top region in 20-layer LFR sample.

**Figure 6:**
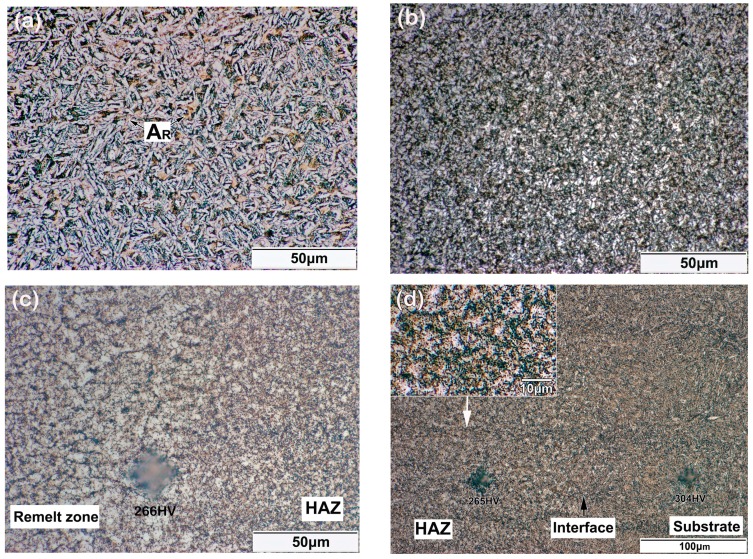
Microstructure of different areas in the 20-layer LFR sample: (**a**) top of the laser repaired layers; (**b**) bottom of the laser repaired layer; (**c**) the interface of the remelt zone and HAZ; and (**d**) the interface of HAZ and substrate.

The morphology of the 20-layer LFR sample is shown in Figure 5, which included laser-repaired layers, remelt zone, HAZ, and substrate. The macroscopic distribution of the 20-layer LFR sample was similar to the five-layer LFR sample. The cross-section micrograph presented in Figure 5 shows good metallurgical fusion between the LFR layers and substrate. The LFR process did not introduce observable defects such as porosity, lack of fusion, or cracks. We inferred that the thickness of the 20-layer LFR was approximately 2.54 mm. The width of HAZ in the 20-layer LFR sample increased slightly to 1.45 mm compared with that of the five-layer LFR sample. Figure 6 shows the highly-magnified morphology of the top region in the 20-layer LFR sample. As can be observed from Figure 6a, the microstructure of the last LFR layer is characterized by mostly bainite and network retained austenite. Nevertheless, the microstructure of the bottom of the laser repaired layers included fine ferrite and carbides. As can be observed from Figure 6c,d, the microstructures of the remelt zone and HAZ were characterized by ferrite and carbides. The most obvious feature in Figure 6c is the grain coarsening of ferrite that occurred at the interface between the remelt zone and the HAZ. The grain size of ferrite in the interface was approximately 5–6 μm, which is about two times of that in the top of the HAZ.

**Figure 7:**
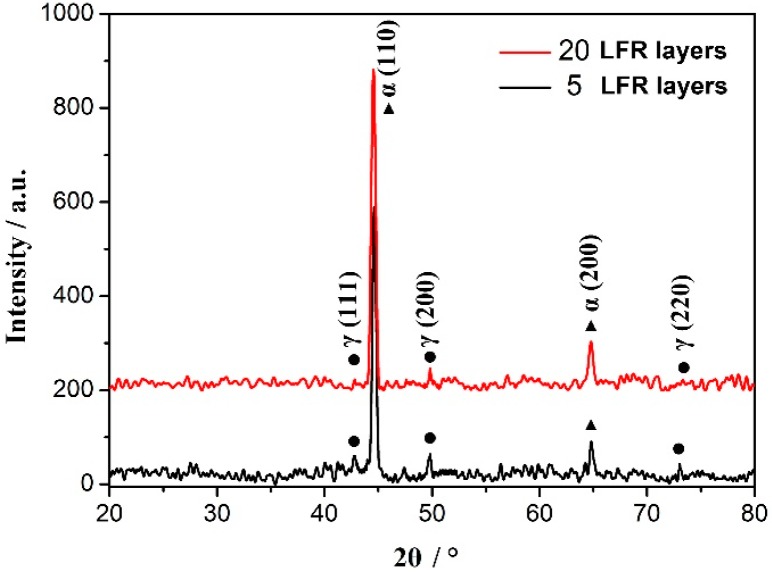
X-ray diffraction (XRD) patterns of the top regions of two kinds of LFR layered samples.

The amount of retained austenite decreased in the last LFR layer of the 20-layer LFR sample compared with that in the five-layer LFR sample, as can be seen in Figure 4a and Figure 6a. In order to confirm the difference, the crystal phases of the last layers in the two LFR samples were identified by X-ray diffraction. The XRD patterns are shown in Figure 7, showing α_B_ and γ peaks arising from different hkl planes and having varying intensities. Bainite peaks were due to the α(110) and α(200) in the two LFR samples. The presented austenite reflections in five-layer LFR sample were (111), (200) and (220), whereas in the 20-layer sample there was (200). With increasing numbers of LFR layers, the amount of retained austenite decreased. The presence of bainite and austenite was in accordance with the OM observations (Figure 4a and Figure 6a).

Under equilibrium processing conditions, upon cooling from the austenite region to below the eutectoid temperature, the prospective microstructure for the given 34CrNiMo6 powder composition (Table 1) should mainly have consisted of ferrite and pearlite. However, the cooling rates for laser single cladding are typically as high as 10^3^ K/s, and this resulted in a deviation from the equilibrium microstructure. According to the Continuous Cooling Transformation (CCT) curve for 34CrNiMo6 steel reported in Huang et al. [22], extremely rapid cooling rates place the laser cladding process to the extreme left hand side of the diagram, below the martensite start line. The cooling curves of 0.038 °C/s and 0.15 °C/s distances on the CCT diagram show that during cooling the austenite transforms to ferrite and pearlite, which is typical for a hypoeutectoid alloy. Martensite would subsequently form, preceded by ferrite and bainite, when the average cooling rate is greater than 2.5 °C/s.

It can be inferred that the heat accumulation of LFR would result in a sharp rise in sample temperature, thereby greatly reducing the cooling rate of the LFR sample. It can be deduced from the microstructure of the last LFR layer, which includes bainite and retained austenite that the average cooling rate is between 0.15 °C/s and 2.5 °C/s. The different microstructures along the LFR direction should result from various thermal histories at different regions during the LFR process. Owing to the lack of recrystallization in the last layer in the multi-layer deposition of the LFR process, the microstructure of some of the last layers differs from that of the other layers. The former laser forming layers can be plotted into the remelt zone (RZ) and HAZ. The HAZ includes the fully austenitic region and tempering region. The same microstructure evolution occurring in the final repair layer, the remelting zone, and the fully austenitizing zone are austenitic to bainite. The tempering effect was not distinct due to the rapid heat dissipation, which only caused the coarsening of carbides and reduction in retained austenite. The microstructural transformation of the repaired sample via LFR from bainite to ferrite can be attributed to the tempering process. The deposition of new layers generated new thermal cycles and consequently, the former laser forming cladding undergoes phase transformations that depend on its microstructure. The tempering temperature and time increase with the development of the LFR process. Tempering above 400 °C transforms bainite into ferrite and cementite.

The microstructure of 34CrNiMo6 steel consists of numerous retained austenites in the last LFR layers that can be attributed to the fast-cooling of the LFR process and the austenite-stabilizing elements within 34CrNiMo6 alloy. With increasing LFR layers, the cooling rate becomes slower and the retained austenite content decreases. Austenite-stabilizing elements include C, Ni, and Mn, which lower the critical temperature and expand the temperature range of the stability of austenite.

### 3.2. Microhardness

**Figure 8:**
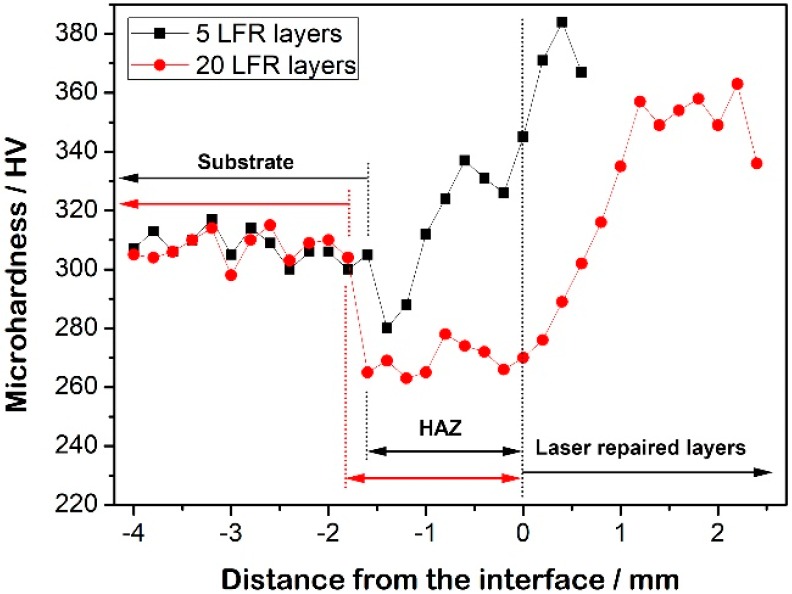
Microhardness distribution across the cross-section of the LFR samples.

Microhardness measurements were taken throughout the top regions of the LFR sample. Figure 8 shows the hardness distribution on the longitudinal section of the five-layer and 20-layer LFR samples. The substrate hardness was 309 HV, which is the hardness of this steel in the quenching and tempering condition. For the five-layer LFR sample, the microhardness of the laser-repaired layers was significantly improved compared to the substrate. The microhardness of the top of the HAZ was higher than that of the substrate, whereas the microhardness of the bottom of HAZ was slightly lower than the hardness of the substrate. For the 20-layer LFR sample, the microhardness of the top of the laser-repaired layers within 1.6 mm of the top surface was significantly improved compared to the substrate. The microhardness values of the bottom of the laser-repaired layers and HAZ were lower than that of the substrate. The 20-layer LFR sample showed lower hardness than that of the five-layer LFR sample due to the thermal accumulation of the LFR process. The minimum hardness of the LFR sample was 263 HV, which appeared in the bottom of HAZ of the 20-layer LFR specimens, corresponding to the ferrite and carbides phase (Figure 6d). With the increase in LFR layers, the cooling rate at the currently deposited layer decreased, and the tempering effect on the already deposited layers became remarkable. Therefore, the ferrite content increased (Figure 6), leading to the decrease in microhardness. The result of hardness distribution in the top region of the LFR sample is consistent with the above microstructural analysis.

Both hardness and strength are the important properties of laser forming repaired samples, but the laser forming repaired samples in this study were insufficient to perform extensive tensile testing. However, hardness testing can be used to estimate tensile properties, particularly ultimate tensile strength [28,29]. Reliable hardness-strength correlations allow for rapid overall tensile property evaluations using hardness testing instead of elaborate tensile testing [30]. In order to determine the relationship between the ultimate tensile strength (UTS) and hardness (HB), a number of relationship were established. In practice, the simplest equation is most often used:

UTS = k × HB,(1)

where k is a coefficient [31]. According to standard ISO 18265, the coefficient k in the range of 3.21 to 3.54 is recommended to use for steel.

It can be seen from Figure 8 that the minimum hardness values of the five-layer LFR sample and the 20-layer LFR sample were 280 HV and 263 HV, respectively. For materials with a hardness less than 450 HB, HB and HV almost coincide. For the range 80 < HV ≤ 250, the equation is HB = HV, whereas for the range 250 < HV ≤ 500, the equation is HB = HV − 0.0002HV (HV − 57) [30]. It can be calculated that the minimum hardness values of the five-layer LFR sample and the 20-layer LFR sample were 268 HB and 252 HB, respectively. According to previous research [22], when the minimum hardness of the laser solid formed 34CrNiMo6 steel was 280 HV, the UTS reached 993 MPa. When the minimum hardness was 280 HV, the UTS was calculated at 949 MPa by selecting k = 3.54, which is smaller than the UTS value obtained by the previous experiment. So, the coefficient k was chosen to be 3.54 in this study. In summary, we inferred that the UTS of the five-layer LFR sample and the 20-layer LFR sample were 993 MPa and 892 MPa, respectively. Zhang et al. [32] tested the tensile properties of forged 34CrNiMo6 steel in the spindle of a wind turbine, whose UTS was 842 MPa. The UTS of wrought 34CrNiMo6 steel in EN 10083-1 standard is not less than 800 MPa. Although the UTS of the LFR samples calculated by the UTS-HB relationship is not completely accurate, it can also be inferred that the UTS of the LFR samples can reach those of the wrought standard.

### 3.3. Tribological Properties

**Figure 9:**
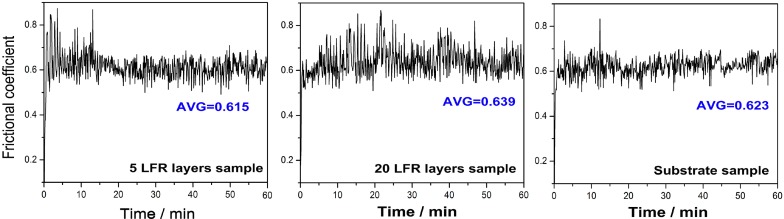
Frictional coefficients of three kinds of sample.

Figure 9 shows the changes in coefficients of friction (COFs) of the repaired samples and substrates against a Si_3_N_4_ ball under dry sliding condition. The average COFs of five-layer LFR, 20-layer LFR, and substrate samples were 0.615, 0.639, and 0.623, respectively. The wear process was divided into running-in period and stable wear period. The running-in time is very short and there is no wear failure period. Among them, the COFs of the five-layer LFR and substrate samples varied a little with the sliding time after 12 min, but the COF of the 20-layer LFR sample obviously fluctuated. The fluctuation of the COF in the 20-layer LFR sample may be caused by the debris that is produced in the wear process. The debris was hard to exhaust from the wear zone. Some of the falling debris was squeezed into the worn zone, causing the fluctuations in the COF. Although there were some differences in detail, the average COFs of the three samples were not much different.

**Figure 10:**
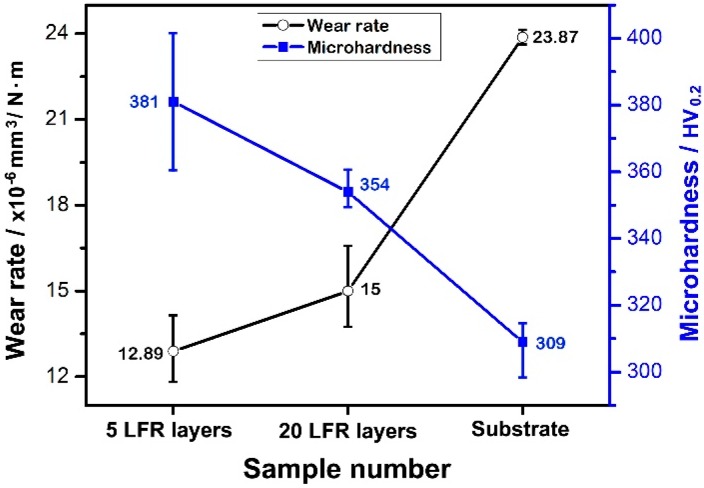
Wear rate and microhardness of three kinds of wear testing samples.

The wear rate is calculated by the following equation [33,34]:

W = V/(S × P) = Δm/(ρ × S × P) [mm^3^/N·m], (2)

where V is the worn volume, P is the normal load, S is the total sliding distance, △m is the wear loss, and ρ is the density of the 34CrNiMo6 steel. Figure 10 shows the wear rates of the three kinds of samples. The wear rates of the five-layer LFR, 20-layer LFR, and substrate samples were 12.89 × 10^−6^, 15 × 10^−6^, and 23.87 × 10^−6^ mm^3^/N·m, respectively. The wear rates of laser forming repaired samples were far less than that of the substrate. Moreover, the five-layer LFR specimen had the lowest wear rate, which was 54% of that of the substrate. The 20-layer LFR sample showed a higher wear rate than that of the five-layer LFR sample. The wear rate increases with increasing LRF layers due to the greater thermal accumulation of the LFR process. It indicated that laser forming repaired samples have better wear resistance compared to the substrate under the repaired layers conditions in this study.

**Figure 11:**
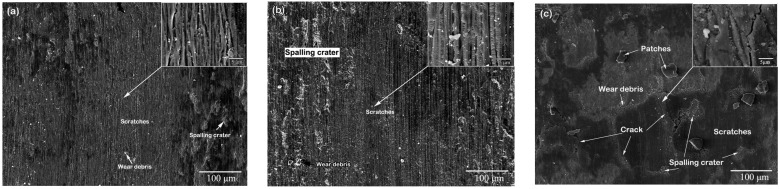
SEM micrographs of the worn surfaces of: (**a**) five-layer LFR; (**b**) 20-layer LFR; and (**c**) substrate.

To further characterize laser forming repair on the tribological behavior of 34CrNiMo6 steel, the captured SEM images of the worn surfaces are provided in Figure 11. The worn surface of the five-layer LFR sample is relatively smooth with slight scratches, small spalling crater, and a small amount of wear debris (Figure 11a), which reveals that slight abrasive wear occurred. The worn surface of the 20-layer LFR sample had more spalling craters and wear debris compared to the five-layer LFR sample (Figure 11b), which indicates that the wear mechanism was abrasive wear. ASTM International defines abrasive wear as the loss of material due to hard particles or hard protuberances that are forced against and move along a solid surface. There were some wear debris, patches, spalling craters, and scratches on the worn surface of the substrate (Figure 11c). Furthermore, long cracks were clear. It was illustrated that the substrate suffered severe abrasive wear and contact fatigue wear. Contact fatigue wear is a process by which the surface of a material is weakened by cyclic loading. Contact fatigue wear is generated when the wear particles are detached by the cyclic crack growth of microcracks on the surface. These microcracks are either superficial cracks or subsurface cracks.

The material properties that affect the friction and wear behavior include hardness, elastic modulus, crystal structure, microstructure, and composition [35]. Khrushchov et al. [36] performed a large amount of testing and found an inverse relationship between wear rate and hardness for steel. The hardness was inversely linearly related to abrasive wear. Ehtemam-Haghighi and colleagues found a positive relationship between wear resistance and hardness for titanium alloy [37,38]. The microhardness of the wear-tested surface of the three kinds of samples is shown in Figure 10. The microhardness values of the five-layer LFR, 20-layer LFR, and substrate samples were 381, 354, and 309 HV, respectively. The hardness of the 20-layer LFR sample was 27 HV lower than the hardness of the five-layer LFR sample. The hardness of the repaired specimen was significantly higher than that of the substrate, which endowed the repaired specimen with excellent wear resistance. Note that the microhardness of the Si_4_N_3_ ceramic ball (1500 HV) was obviously higher than wear-tested samples. Therefore, the wear mainly occurred in the tested samples, rather than in the ceramic ball.

**Figure 12:**
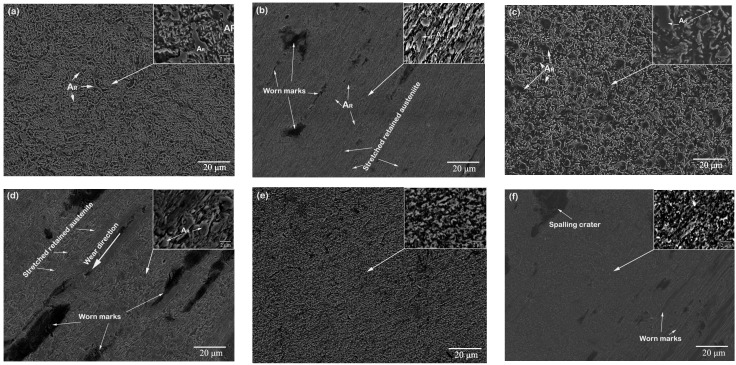
SEM micrographs of sub-surface material showing microstructural changes (**a**,**c**,**e**) before and (**b**,**d**,**f**) after wear test: (**a**,**b**) five-layer LFR, (**c**,**d**) 20-layer LFR, and (**e**,**f**) substrate.

Microstructure is also important for tribological properties. Because of the higher strain-hardening capacity and ductility, austenite and bainite of equal hardness are more wear resistant than ferrite, pearlite, or martensite [35]. Figure 12 shows the microstructure details of the worn subsurface of the three kinds of wear testing samples before and after wear testing. The microstructure of the laser repaired layer included bainite and dispersed retained austenite (Figure 12a,c), whereas the microstructure of the substrate was tempered martensite. After the wear process, the retained austenite of the laser repaired layers was reduced and stretched (Figure 12b,d). Plasticity-dominated processes due to the stretching of the retained austenite play an important role in the increase in the wear resistance. Some retained austenite in the surface of the wear-testing sample can transform into bainite during the wear process. The bainite and retained austenite were further refined. As part of the friction energy was absorbed for the transformation of retained austenite and refinement of the microstructure, less energy was available for nucleation and propagation of subsurface cracks [39]. The wear process had no significant effect on the microstructure of the substrate, which indicated that both hardness and microstructure contribute to improving the tribological properties of the laser forming repaired samples.

## 4. Conclusions

In this study, 34CrNiMo6 repaired layers were successfully deposited using laser forming repair on the 34CrNiMo6 substrate with no cracks or porosity, which were metallurgically combined with the substrate. The laser forming repaired sample could be divided into four zones, including laser repaired layers, remelt zone, heat affected zone, and substrate. When the repair layers were small (five layers), the microstructure of the laser repaired layers included bainite and retained austenite. When the repair layers increased to 20 layers, the microstructure in the top of the laser repaired layers was still bainite and retained austenite, but the microstructure in the bottom of the laser repaired layers transformed into ferrite and cementite.

The microhardness of the five-layer LFR was is higher than that of the substrate, except for a small area in the bottom of the HAZ. The microhardness of the 20-layer LFR sample within 1.6 mm of the top surface significantly improved compared to the substrate. The minimum hardnesses of the five-layer LFR and 20-layer LFR sample were 280 and 263 HV, respectively. Through the hardness-strength relationship, we inferred that the UTS of the LFR samples can reach those of the wrought standard. The average COFs of five-layer LFR, 20-layer LFR, and substrate samples were 0.615, 0.639, and 0.623, respectively. Among them, the average COF of the five-layer LFR sample was the smallest. The wear rates of five-layer LFR, 20-layer LFR, and substrate samples were 12.89 × 10^−6^, 15 × 10^−6^, and 23.87 × 10^−6^ mm^3^/N·m, respectively. The laser forming repaired samples had better wear resistance compared to the substrate, attributed to their hardness and microstructure. The wear mechanism of laser forming repaired samples was abrasive wear, whereas that of the substrate is abrasive wear and fatigue wear.

## Figures and Tables

**Table 1:** Chemical compositions of 34CrNiMo6 powder (wt %).

Element	Content (wt %)
C	0.30–0.38
Cr	1.3–1.7
Ni	1.3–1.7
Mo	0.15–0.30
Mn	0.50–0.80
Si	0.20–0.40
Fe	Balance

**Table 2:** Processing parameters of laser forming repair for 34CrNiMo6 steel.

Laser Powder (W)	Spot Diameter (mm)	Increase in Axis-Z (mm)	Overlap (%)	Scanning Speed (mm·min^−1^)	Powder Feeding Rate (g·min^−1^)
2800	2.5	0.2	45	600	10–15
